# Hematemesis, a very rare presentation of solid pseudo-papillary tumors of the pancreas: a case report

**DOI:** 10.1186/1752-1947-2-271

**Published:** 2008-08-13

**Authors:** Stylianos Apostolidis, Theodossis S Papavramidis, Akis Zatagias, Antonis Michalopoulos, Vassilis N Papadopoulos, Daniel Paramythiotis, Nick Harlaftis

**Affiliations:** 1 1st Propedeutic Surgical Department, A.H.E.P.A. University Hospital of Thessaloniki, Aristotle's University of Thessaloniki, Thessaloniki, Greece

## Abstract

**Introduction:**

Solid pseudo-papillary tumors of the pancreas are rare and typically present in young female patients. They are slowly growing masses that may attain large size, and are of low malignant potential. Surgical resection is usually curative.

**Case presentation:**

A 71-year-old woman presented to the emergency department with an episode of hematemesis but was otherwise hemodynamically stable. Emergency gastroscopy revealed a bleeding mass projecting to the duodenum. Fluid, blood and electrolyte resuscitation followed. Computed tomography revealed a small mass in the head of the pancreas. A Whipple operation was performed. Pathology revealed a solid pseudo-papillary tumor. The postoperative course of the patient was uneventful and no recurrence was present a year after the operation.

**Conclusion:**

In our case, the most noteworthy observations concern the small size of the tumor, the age of the patient and the presenting symptom. However, pancreaticoduodenectomy in a 71-year-old woman is a major effort and should only be undertaken by centers and surgeons experienced in complex hepatobiliary surgery. Furthermore, the unique nature of this case reminds every clinician that each patient has to be considered separately and with extreme caution.

## Introduction

Solid pseudo-papillary tumor (SPT) is a rare low-grade malignancy of the pancreas, accounting for 0.2% to 2.7% of the primary tumors of the organ [1]. The largest review of SPTs reports only 718 cases in the English literature [2]. SPTs of the pancreas are typically present in young female patients. They are slow-growing masses that may attain large size, and are of low malignant potential. In most cases (~80%), SPTs present either as a mass or as pain, while the tumor is asymptomatic in about 15% of the cases [2]. Surgical resection is usually curative [1,2]. This case is extremely interesting because it concerns a small SPT of the head of the pancreas in an aged patient who presented with an episode of hematemesis.

## Case presentation

**Figure 1:**
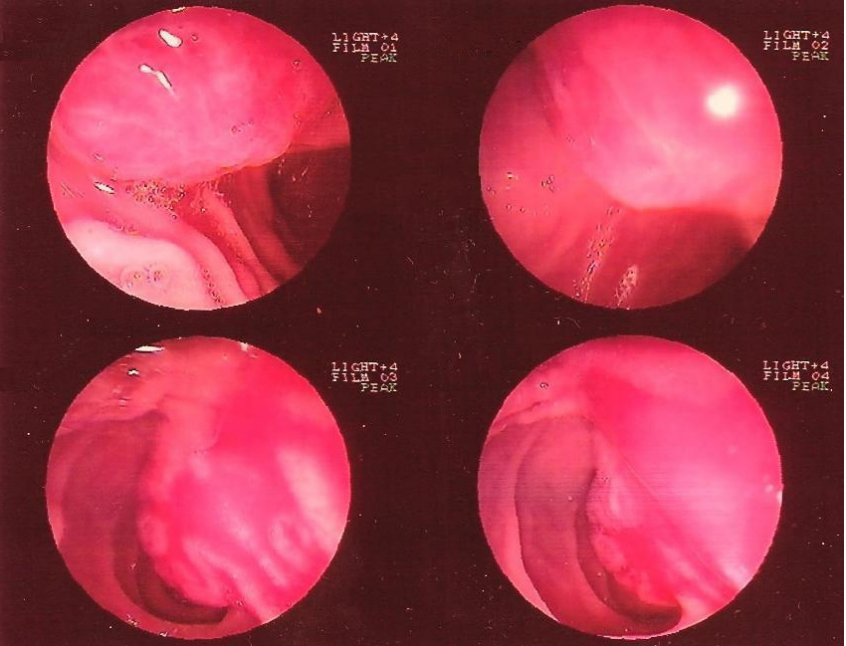
**Upper gastrointestinal tract endoscopy revealing a small (about 2 cm), slightly bleeding mass projecting to the duodenum**.

A 71-year-old woman presented to the emergency department with a single episode of hematemesis but was otherwise hemodynamically stable. Hematocrit was 23% with hemoglobin at 7.6 mg/dl. Fluid, blood and electrolyte resuscitation followed. Emergency endoscopy of the upper gastrointestinal tract revealed a small (about 2 cm), slightly bleeding mass projecting to the duodenum (Fig. 1). The next day, computed tomography (CT) revealed a small mass measuring 0.4 × 0.4 × 3 cm in the head of the pancreas. Ten days after the CT, a typical Whipple operation was performed. The surgical specimen included a bluish mass localized in the head of the pancreas. Pathology and immunohistochemistry of the tumor revealed a SPT. The postoperative course of the patient was uneventful and she was discharged at the 13th postoperative day. A year after the operation, no recurrence is present.

## Discussion

SPT is a very uncommon pancreatic tumor that affects mainly women (F/M 9.78:1) [2]. The mean age of its appearance is 21.97 years (ranging from 2 to 85 years) [2]. Most of the patients are young (~22% are younger than 18 years), but a considerable 6% of the cases are older than 51 years [2]. To our knowledge, there are only 7 cases (including this case) of SPTs presenting in patients older than 70 years.

SPT generally becomes clinically evident as a palpable mass at the epigastrium, the left or the right hypochondrium [2]. Furthermore, pain can also be the first sign of a SPT [2]. To our knowledge, this is the first case where the first sign of SPT is hematemesis. Despite technical advances, pre-operative diagnosis is difficult.

Concerning the surgical approach, a great number of techniques are employed. The low grade of malignancy of this tumor has led some surgeons to perform simple enucleation of the neoplasm [2]. However, distal pancreatectomy with splenic preservation or pancreatoduodenectomy, depending on the location of the tumor, represent the procedures of choice [2]. The mean size of SPTs is 6.08 cm, so the tumor in this case is classified as small.

## Conclusion

In our case, the most noteworthy observations concern the small size of the tumor, the age of the patient and the presenting symptom. However, pancreaticoduodenectomy in a 71-year-old woman is a major effort and should only be undertaken by centers and surgeons experienced in complex hepatobiliary surgery. Furthermore, the unique nature of this case reminds every clinician that each patient has to be considered separately and with extreme caution.

## Competing interests

The authors declare that they have no competing interests.

## Authors' contributions

SA was the main surgeon and was involved in revising the draft critically for important intellectual content. TSP received the patient to the emergency department, was advising doctor and was involved in drafting the manuscript and revising it critically for important intellectual content. AZ, AM and VNP were auxiliary surgeons and were involved in revising the draft critically for important intellectual content. DP received the patient to the emergency department and was involved in drafting the manuscript. NH carried out strategic planning for the treatment of the patient and was involved in revising the draft critically for important intellectual content. All authors have given final approval of the version to be published.

## Consent

Written informed consent was obtained from the patient for publication of this case report and any accompanying images. A copy of the written consent is available for review by the Editor-in-Chief of this journal.
